# Supplementary material for: A bilateral filtering-based image enhancement for Alzheimer disease classification using CNN
Source: PLoS One. 2024 Apr 19;19(4):e0302358. doi: 10.1371/journal.pone.0302358 (PMC11029622; doi:10.1371/journal.pone.0302358)
Supplement: S1 Checklist — (PDF) [file pone.0302358.s001.pdf]

PLOS ONE Clinical Studies Checklist

***PLOS ONE* manuscript number:** ______PONE-D-23-12558______

Complete the following if your study involved human participants or human subjects' data. These questions should be addressed for prospective and retrospective studies.

1. Did you obtain ethics approval for this study?

   - If yes, please upload (file type "Other") the original approval document you received from your ethics committee. If the original document is in another language, please also provide an English translation.

     N/A

   - If you did not obtain ethical approval, please explain why this was not required.

No approval was required. Human subjects' data were obtained from a restricted online data repository (adni.loni.usc.edu) instead of human participants. Access to this dataset is contingent upon compliance with the ADNI Data Use Agreement and the publication's policies, which govern the ethical use of their datasets. Once approval has been granted, we believe ethical consideration has been given to the research and data.

Again, departmental faculty members reviewed the research, as this is part of my doctoral dissertation. We believe that ethical consideration was given to the work after it was approved.

2. If your study involved human participants, please report in the Methods section when participants were recruited to the study.

N/A: No human participants were involved in the study.

3. If you are reporting a study of medical records or archived samples, please report in the Methods section the date range in which human subjects' data/samples were collected and the date(s) when you conducted this study.

   Completed

4. Please specify in the Methods section whether authors had access to information that could identify individual participants during or after data collection.

   N/A: Authors had no access to such information

5. If you are reporting an observational study – i.e. cohort, case-control, and cross-sectional studies – we recommend that the work is reported as per the requirements of the STROBE guidelines, and that you provide a completed STROBE checklist as a Supporting Information file with your submission.

   The STROBE checklist was developed to improve the reporting of observational human subjects research, and is available here: http://strobe-

statement.org/fileadmin/Strobe/uploads/checklists/STROBE_checklist_v4_combined_PlosMedicine.docx.

N/A

6. Please ensure that the author list and Corresponding Author entered in Editorial Manager match the author list and Corresponding Author in your manuscript file.

___ **Completed**
