# Supplementary material for: A bilateral filtering-based image enhancement for Alzheimer disease classification using CNN
Source: PLoS One. 2024 Apr 19;19(4):e0302358. doi: 10.1371/journal.pone.0302358 (PMC11029622; doi:10.1371/journal.pone.0302358)
Supplement: Attachment — Submitted filename: Response to reviewers.pdf [file pone.0302358.s002.pdf]

UNIVERSITY OF ENERGY AND NATURAL RESOURCES, SUNYANI
Department of Computer Science and Informatics
Post Office Box 214, Sunyani, Ghana
Telephone: +233 (0) 247242882 / +233 (0) 501347618
Email: nicodemus.awarayi@uenr.edu.gh
MR. NICODEMUS SONGOSE AWARAYI

30th June, 2023.

The Editor

PLOS ONE Journal

Dear Sir,

Response to Review Comments

I am writing to express our appreciation to the editors and reviewers for constructively critiquing our manuscript and providing comments necessary for improving it. In this letter, I would like to state that all comments have been addressed and the manuscript has been modified accordingly. All major modifications are highlighted in yellow for easy tracking. The responses to the review's comments are presented as follows:

Manuscript Number: PONE-D-23-12558

Title: A Bilateral Filtering-based Image Enhancement for Alzheimer Disease Classification Using CNN

Editor comments #3, 4: Data availability

Response: The datasets used in this study were obtained from the Alzheimer's Disease Neuroimaging Initiative (ADNI) database, a public data repository with access control. Researchers can apply to be granted access to the datasets. Due to the data use policy of the ADNI, the authors do not have absolute control over the data; however, we have duly cited the data source.

The authors would like to state that we do not have control over the dataset due to the data use policy restrictions of the ADNI; however, we will include the link to the database, to which researchers can submit requests to be granted access by the ADNI

Reviewer 1, Comment # 1: The abstract must be re-written, focusing on the technical aspects of the proposed model, the main experimental results, and the metrics used in the evaluation. Briefly discuss how the proposed model is superior.

Response: Thank you for your comments and suggestions. The abstract has been rewritten, taking into account all the suggestions made by the reviewer.

Authors action:

The purpose of this study is to develop an optimally performing convolutional neural network to classify Alzheimer disease into mild cognitive impairment, normal controls, or Alzheimer disease classes using a magnetic resonance imaging dataset. To achieve this, we focused the study on addressing the challenge of image noise, which impacts the performance of the deep learning model. The study introduced a scheme for enhancing images so as to improve the quality of the

datasets. Specifically, an image enhancement algorithm based on histogram equalization and bilateral filtering techniques was deployed to reduce image noise and improve image quality. Subsequently, a convolutional neural network model comprising four convolutional layers and two hidden layers was devised for classifying Alzheimer disease into three (3) distinct categories, namely mild cognitive impairment, Alzheimer's disease, and normal controls. The model was trained and evaluated using a 10-fold cross-validation sampling approach with a learning rate of 0.001 and 200 training epochs at each instance. The proposed model yielded notable results, such as an accuracy of 93.45% and an area under the curve value of 0.99 when trained on the three classes. The model further showed superior results on the binary classification of Alzheimer disease versus normal controls, Alzheimer disease versus mild cognitive impairment, and mild cognitive impairment versus normal controls, resulting in accuracies of 94.39%, 94.92%, and 95.62%, respectively.

Reviewer 1, Comment # 2: Additionally, method names should not be capitalized. Moreover, it is not the best practice to employ abbreviations in the abstract, they should be used when the term is introduced for the first time

Response: Your comments are duly appreciated: The abbreviations in the abstract have been written in full. Please refer to the abstract section (page 1).

Reviewer 1, Comment # 3,4 and 5: The contribution of the current study must be briefly discussed as bullet points in the introduction. And motivation must also be discussed in the manuscript. Introduction section in too lengthy, some part of the introduction can be moved as the literature review. Introduction section must discuss the technical gaps associated with the current problem.

Response: Thank you for your contribution. The introduction has been modified in accordance to the reviewer's comments. The literature was imbedded in the introduction making it too lengthy. The literature review in the introduction has, therefore, been moved to a new section (page 2-4). The motivation and technical gaps have been expatiated (page 1).

Authors action:

This is a major concern that requires much attention, as a deep learning model's performance highly depends on the volume and quality of the datasets. In medical imaging, particularly Alzheimer's disease, this continues to be a challenge, though various research institutions continue to make data available. As the data is collected over a period of time, there is a tendency for the datasets to not be enough for training models, which can affect the performance of the training, leading to overfitting or poor generalizability of the model. Again, when the data is noisy, it highly affects the accuracy of the model's prediction, as the noise can introduce false labels into the datasets, thereby affecting the reliability of the model. MRI images are highly susceptible to noise due to the varying image scanning protocols, imaging artifacts, and moving artifacts being used. Image quality can also vary as a result of the different imaging equipment being used. To address these challenges, it requires developing more robust deep learning models that are more tolerant

to noisy images [13, 14] or applying a data preprocessing approach to addressing image quality [15-18].

The motivation of our study is to propose a more reliable approach to improving image quality with ease for classifying Alzheimer's disease with improved accuracy. As such, the research devised a proficient approach for improving image quality through the utilization of bilateral filtering and histogram equalization techniques. The proposed method aimed to reduce noise and enhance brightness in images, thereby improving the quality of images utilized in categorizing AD. The primary contribution of this study comprises two key components:

- First, an image enhancement technique that effectively reduces noise in MRI images while preserving image quality. An image enhancement algorithm for color images was developed using two image processing techniques: image equalization and bilateral filtering. The algorithm mainly improves the quality of the images while maintaining their edges and details.
- Second, a CNN model consisting of four convolutional layers and two hidden layers successfully classifies Alzheimer's disease with greater accuracy than existing deep learning models.

Reviewer 1, Comment #6: The literature section is missing. Authors are recommended to incorporate the same for better comprehensibility of the study.

Response: The literature review was added to the introduction and has been moved into a new section for more clarity. See page 2-4.

Reviewer 1, Comment #7: Authors may provide the architecture/block diagram of the proposed model for better comprehensibility of the proposed model concerning various aspects of the proposed model.

Response: Your suggestions are duly acknowledged. The block diagram has been incorporated for better clarity.

Authors actions:

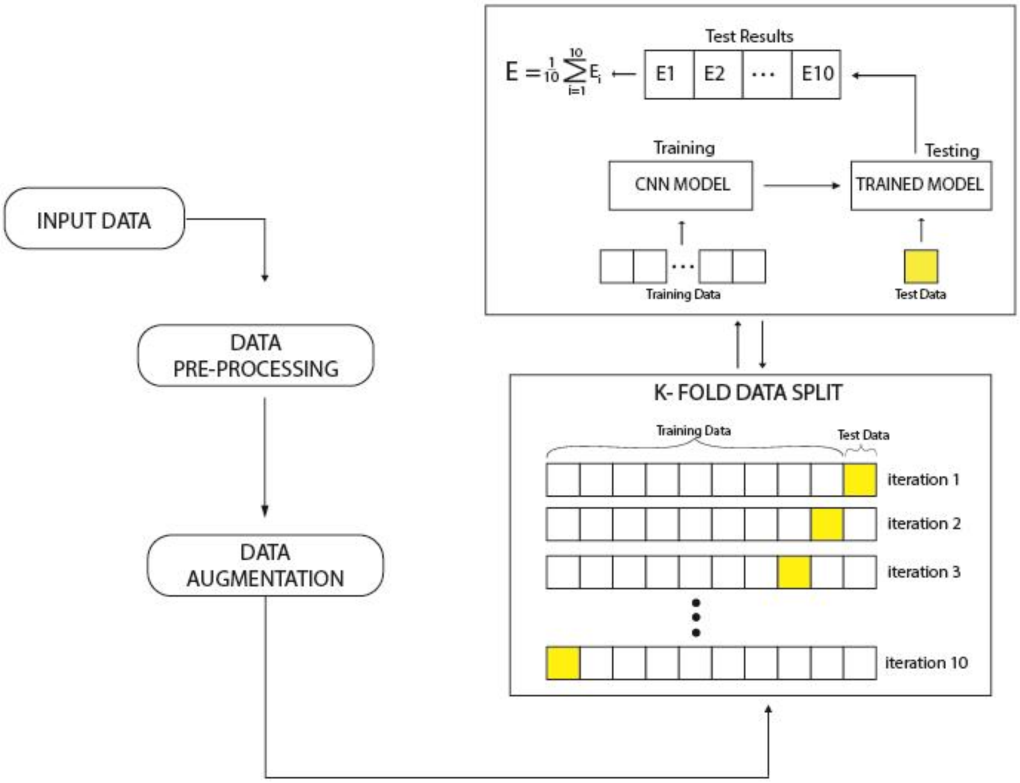

Figure 1. The proposed method defining the flow of activities.

Reviewer 1, Comment #8: What is the size of the input image that is considered for processing and the size of the kernels? Authors may refer and include the content shown in https://doi.org/10.1038/s41598-022-25089-2 for better comprehensibility.

Response: Thank you for the comments. The size of the input images is defined under the datasets section (page 5) and the convolutional neural network model section (page 7). The kernel filter size is also indicated under the convolutional neural network model section (page 7) and the hyperparameters section (page 8).

Authors action:

…Each of the images was initially dimensioned as 256 x 256 pixels 150 and was resized to 64 x 64 pixels.

Reviewer 1, Comment #9: Whether authors have used Stride 1 or Stride, 2 must be presented.

Response: Stride 1 was used in the convolutional layers and has now been included in the manuscript.

Authors action:

All convolutional layers had a stride of one (1), and same padding was employed to keep the dimensions of the output feature map the same as the input picture dimension.

Reviewer 1, Comment #10: For how many epochs does the proposed model execute. what is the initial learning rate, and after how many epochs does the model's learning rqate saturated.

Response: Thank you for the comments. The model was trained for 200 epochs with a learning rate of 0.001, which is incorporated under the hyperparameters section. This has been indicated under various sections in the manuscript (Pages 1, 8, and 9)

Reviewer 1, Comment #11: Majority of the figures lack the clarity; they quality is fair but they must be explained in the text and the figures must be cited.

Response: The figure labeling has been revised, and all figures have been cited appropriately for better clarity.

Reviewer 1, Comment #12: By considering the current form of the conclusion section, it is hard to understand by PlosOne Journal readers. It should be extended with new sentences about the necessity and contributions of the study by considering the authors' opinions about the experimental results derived from some other well-known objective evaluation values if it is possible.

Response: Thank you for the comments. The conclusion has been rewritten for better comprehension. (see page 11).

Authors action:

Alzheimer's disease is a neurological disorder that primarily affects older individuals and currently lacks a definitive cure. Detecting Alzheimer's disease is crucial to minimizing its progression from a mild stage to a chronic stage. Detecting Alzheimer's disease using a deep learning approach mostly comes with numerous challenges, of which the challenge of image quality was addressed in this study. The study proposed and implemented an image quality scheme using histogram equalization and bilateral filtering to improve the quality of the dataset, which was then used to learn the proposed convolutional neural network. The proposed model consisted of four convolutional layers and two fully connected layers. It was trained using 10-fold cross-validation on three class labels, including Alzheimer disease, mild cognitive impairment, and normal controls. The resulting accuracy, precision, recall score, and AUC score stood at 93.45%, 93.70%, 93.24%, and 0.99, respectively. These scores were found to be comparatively higher than those achieved by some selected state-of-the-art existing models.

Reviewer 1, Comment #13: English proofreading is strongly recommended for a better understanding of the study, and the quality of the figures must be tremendously improved.

Response: Thank you for the recommendation. Proofreading has been done.

Reviewer 1, Comment #14: Captions of the Figures not self-explanatory. The caption of figures should be self-explanatory, and clearly explaining the figure. Extend the description of the mentioned figures to make them self-explanatory. For example: Fig 1. Proposed CNN architecture.

Response: The figure labeling has been revised, and all figures have been cited appropriately for better clarity.

Authors action: Fig 2. The proposed CNN model for classifying Alzheimer's disease.

Reviewer 2, Comment #1,2: Concept addressed in this study is quite good. The contribution of the current study must be briefly discussed as bullet points in the introduction. And motivation must also be discussed in the introduction of the manuscript. The introduction and the literature must be tremendously improvised with a focus on the limitations of existing models

Response: Thank you for your contribution. The introduction and the literature review have been modified in accordance with the reviewer's comments. The literature review in the introduction has been moved to a new section (pages 2–4) for better clarity. The motivation and technical gaps have been explained (page 1).

Authors action:

This is a major concern that requires much attention, as a deep learning model's performance highly depends on the volume and quality of the datasets. In medical imaging, particularly Alzheimer's disease, this continues to be a challenge, though various research institutions continue to make data available. As the data is collected over a period of time, there is a tendency for the datasets to not be enough for training models, which can affect the performance of the training, leading to overfitting or poor generalizability of the model. Again, when the data is noisy, it highly affects the accuracy of the model's prediction, as the noise can introduce false labels into the datasets, thereby affecting the reliability of the model. MRI images are highly susceptible to noise due to the varying image scanning protocols, imaging artifacts, and moving artifacts being used. Image quality can also vary as a result of the different imaging equipment being used. To address these challenges, it requires developing more robust deep learning models that are more tolerant to noisy images [13, 14] or applying a data preprocessing approach to addressing image quality [15-18].

The motivation of our study is to propose a more reliable approach to improving image quality with ease for classifying Alzheimer's disease with improved accuracy. As such, the research devised a proficient approach for improving image quality through the utilization of bilateral filtering and histogram equalization techniques. The proposed method aimed to reduce noise and enhance brightness in images, thereby improving the quality of images utilized in categorizing AD. The primary contribution of this study comprises two key components:

- First, an image enhancement technique that effectively reduces noise in MRI images while preserving image quality. An image enhancement algorithm for color images was developed using two image processing techniques: image equalization and bilateral

filtering. The algorithm mainly improves the quality of the images while maintaining their edges and details.

- Second, a CNN model consisting of four convolutional layers and two hidden layers successfully classifies Alzheimer's disease with greater accuracy than existing deep learning models.

Literature review: See page 2-4.

Reviewer 2, Comment #3: Where is the graph for testing loss and accuracy presented in the study?

Response: The authors excluded the graphs because the method employed in the training (10-fold cross validation) generated about 10 loss and 10 accuracy graphs, which we thought would be too many to be presented in the manuscript. The training of the model involved 10 iterations, generating a loss and accuracy graph per iteration.

Reviewer 2, Comment #4: Please discuss more on the implementation platform and the dataset details as two sub-sections in the manuscript.

Response: Your comments are duly appreciated. The datasets have been further described under the dataset section, while the implementation platform is presented under the method section.

Authors action:

…The images collected and used in the experiment, included 1,581 Alzheimer's disease (AD) images, 1,310 mild cognitive impairment (MCI) images, and 1,591 healthy/normal control (NC) images. Each of the images was initially dimensioned as 256 x 256 pixels and later resized to 64 x 64 pixels.

…The study utilized an NVIDIA GeForce GTX 1060 Graphic Processing Unit (GPU) machine operating on CUDA 10.1 with 8 GB of memory allocation to implement and run the model. The model was implemented in the Python programming language using the TensorFlow framework. The various libraries used include Sklearn, Numpy, Keras, Pillow, and OpenCV-Python.

Reviewer 2, Comment #5: Include the block diagram for the proposed approach and its process.

Response: Thank you for the suggestion. The block diagram has been incorporated for better clarity.

Authors actions:

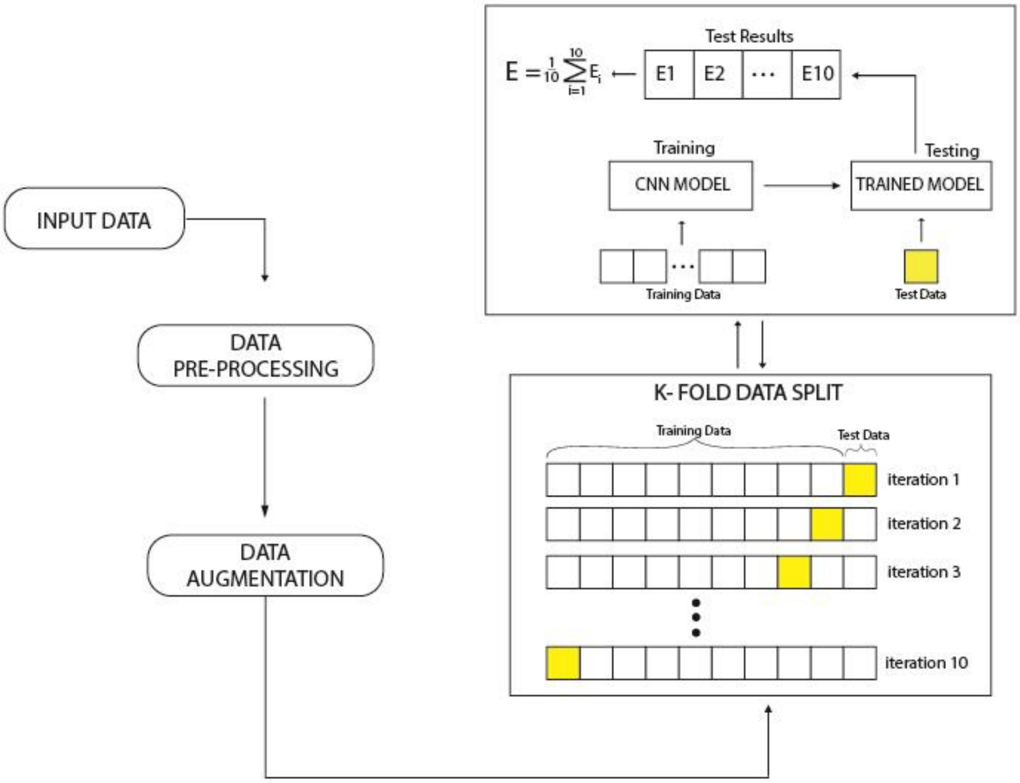

Figure 1. The proposed method defining the flow of activities.

Reviewer 2, Comment #6: By considering the current form of the conclusion section, it is hard to understand by the Journal readers. It should be extended with new sentences about the necessity and contributions of the study by considering the authors' opinions about the experimental results derived from some other well-known objective evaluation values if it is possible.

Response: Thank you for the comments. The conclusion has been rewritten for better comprehension. (see page 11).

Authors action:

Alzheimer's disease is a neurological disorder that primarily affects older individuals and currently lacks a definitive cure. Detecting Alzheimer's disease is crucial to minimizing its progression from a mild stage to a chronic stage. Detecting Alzheimer's disease using a deep learning approach mostly comes with numerous challenges, of which the challenge of image quality was addressed in this study. The study proposed and implemented an image quality scheme using histogram equalization and bilateral filtering to improve the quality of the dataset, which was then used to learn the proposed convolutional neural network. The proposed model consisted of four convolutional layers and two fully connected layers. It was trained using 10-fold cross-validation on three class labels, including Alzheimer disease, mild cognitive impairment, and normal controls. The resulting accuracy, precision, recall score, and AUC score stood at 93.45%, 93.70%,

93.24%, and 0.99, respectively. These scores were found to be comparatively higher than those achieved by some selected state-of-the-art existing models.

Thank you.

Yours sincerely,

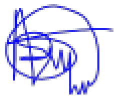

Mr. Nicodemus Songose Awarayi
Corresponding Author
nicodemus.awarayi@uenr.edu.gh
+233247242882
