# Supplementary material for: A bilateral filtering-based image enhancement for Alzheimer disease classification using CNN
Source: PLoS One. 2024 Apr 19;19(4):e0302358. doi: 10.1371/journal.pone.0302358 (PMC11029622; doi:10.1371/journal.pone.0302358)
Supplement: Attachment — Submitted filename: Response to reviewers.pdf [file pone.0302358.s003.pdf]

23rd October, 2023.

The Editor

PLOS ONE Journal

Dear Sir,

Response to Review Comments

I wish to extend my gratitude to the editors and reviewers for their insightful feedback on our manuscript. We have diligently addressed all the comments and made the necessary changes to the manuscript. For your convenience, major revisions have been highlighted in yellow. Detailed responses to the reviewers' comments are provided below:

Manuscript Number: PONE-D-23-12558

Title: A Bilateral Filtering-based Image Enhancement for Alzheimer's Disease Classification Using CNN

Reviewer 3, Comment # 1: There are several grammatical mistakes and typos that must be corrected with careful revision, such as from where this parenthesis "….. [1].)." Is started, and so on.

Response: Thank you for your comments and suggestions. The grammatical mistakes and typos have been rectified. The manuscript has been thoroughly reviewed, and the errors duly corrected. We also used the Grammarly software to check and correct the mistakes.

Reviewer 3, Comment # 2: Most of the abbreviations are incorrect such as Alzheimer's 8 disease, and so on. Every term should be defined completely for the first time and then use the abbreviation throughout the article, do not repeat.

Response: Your comments are duly appreciated. The corrections have been made to that effect.

Reviewer 3, Comment # 3: The first sentence "First, an image enhancement technique that effectively reduces noise in MRI 49 images while preserving image quality" of the contribution seems incomplete. Please revise. Additionally, the contribution is very limited.

Response: Thanks for the comments. The manuscript has been modified accordingly.

Action: The primary contribution of this study comprises two key components:

- First, an image enhancement technique that effectively reduces noise in MRI images while preserving image quality was designed and implemented. The image enhancement algorithm for color images was developed using two image processing techniques: image equalization and bilateral filtering. The algorithm mainly improves the quality of the images while maintaining their edges and details. The bilateral filtering was used to keep the edges and details of the image, while histogram equalization modifies the intensities of the image to improve its contrast.

- Second, a CNN model consisting of four convolutional layers and two hidden layers successfully classifies AD more accurately than existing deep learning models. The model was trained using the k-fold cross-validation technique, ensuring that all the images were effectively used in training and testing the model.

Reviewer 3, Comment # 4: There is no paper organization, please add at the end of the introduction section.

Response: The paper organization has been added at the end of the introduction.

Action: The remaining sections of the paper are the literature review, materials and methods, results, and discussion. The literature review section presents a review of some existing state-of-the-art studies. A description of the dataset, the proposed method, and a detailed description of the experiment are presented under the materials and methods section. The results section reveals the study's experimental results, which are further discussed in the discussion section.

Reviewer 3, Comment # 5 & 6: Generally, the English writing of this article is very low standard. Moreover, the technical depth of the paper is not adequate too. I strictly recommend the authors to have a look at some standard article and learn about the article writing and organization, etc.

Response: Your comment is duly acknowledged. The manuscript has been reviewed to improve the writing standard. A reference was made to Tufail et al. (2023), Allada et al. (2023) and the manuscript writing guideline of the PLOS ONE journal to gather insight to improve the manuscript.

Reviewer 3, Comment # 7: Table 1 summarize the existing CNN model used for AD, although there several more latest models such as "3D Convolutional Neural Networks Based Multiclass Classification of Alzheimer's and Parkinson's Diseases using PET and SPECT Neuroimaging Modalities, Brain Informatics, 2021", "On Improved 3D-CNN Based Binary and Multiclass Classification of Alzheimer's Disease Using Neuroimaging Modalities and Data Augmentation Methods", Journal of Healthcare Engineering, 2021", "Early-Stage Alzheimer's Disease Categorization using PET Neuroimaging Modality and Convolutional Neural Networks in the 2D and 3D Domains", Sensors, 2022", "On Disharmony in Batch Normalization and Dropout Methods for Early Categorization of Alzheimer's Disease" Sustainability, 2022". Beside this, the authors can also refer to more latest models for better comparison and understanding.

Response: Thanks for your recommendations: We have updated Table 1 with more latest models such as Hridhee et al., (2023), Allada et al., (2023), Gowhar et al., (2023) and Tufail et al., (2022).

Reviewer 3, Comment # 8: Figure 1 should be redesigned. Most of the arrows are not correctly connected. Also, keep the figures text always consistent with the paper body text.

Response: Thanks for the observation and suggestion. Figure 1 has been redesigned to ensure the arrows are well connected, and the text is consistent with the paper's body text.

Action:

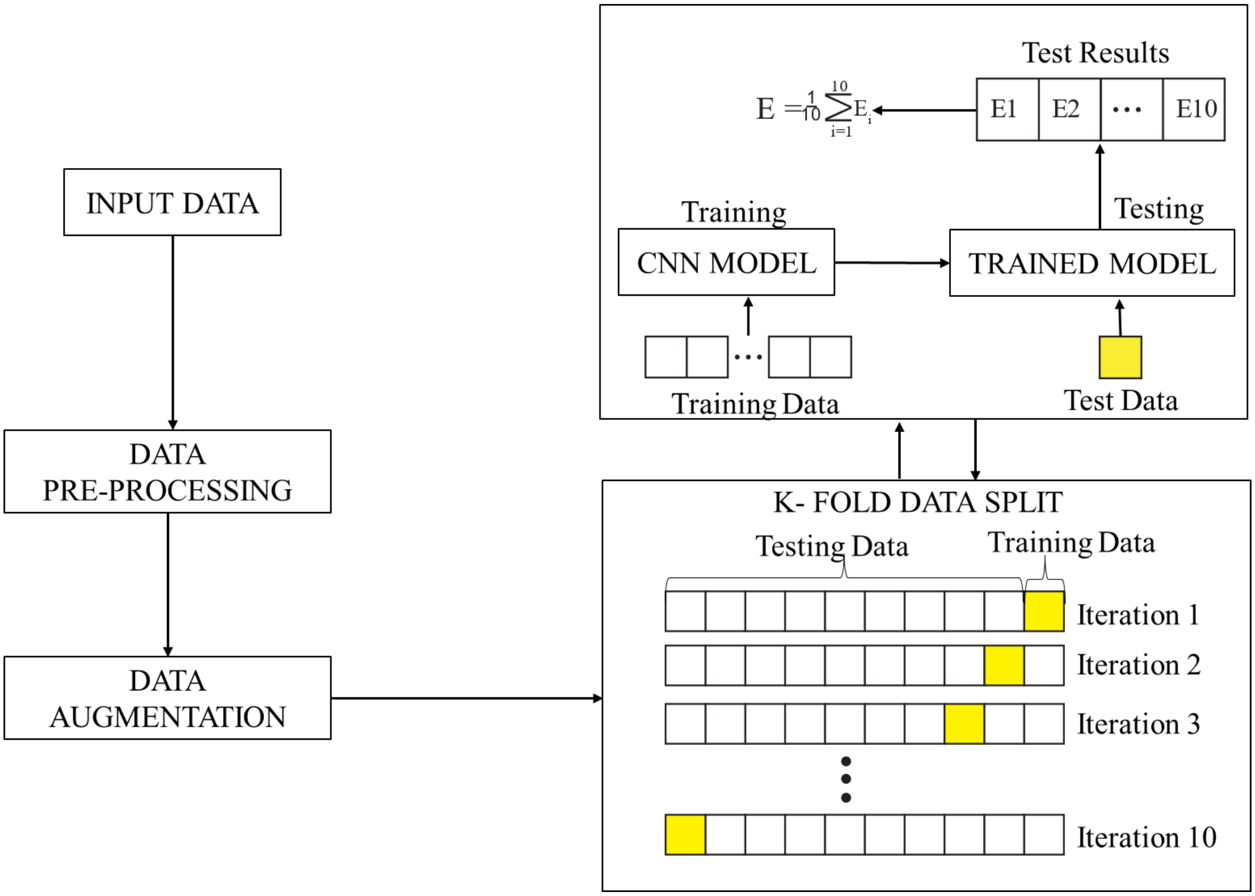

Reviewer 3, Comment # 9: After any equation, in term "Where" w should be small always.

Response: Thanks for the correction. The correction has been done.

Reviewer 3, Comment # 10: Table 4 presents a Comparative analysis of existing and proposed model but the authors should add more latest models in comparison.

Response: Thank you for the recommendation. Latest models, such as Hridhee et al. (2023), Allada et al. (2023), Gowhar et al. (2023) and Tufail et al. (2022) have been added.

Reviewer 3, Comment # 11: Most of the reference are old enough and also limited. Further latest references can be added.

Response: Thank you for the comment. The suggestion has been implemented.

Action:

1. Hridhee RA, Bhowmik B, Hossain QD. Alzheimer's Disease Classification From 2D MRI Brain Scans Using Convolutional Neural Networks. 3rd Int Conf Electr Comput Commun Eng ECCE 2023. 2023;(August):1–6.

2. Allada A, Bhavani R, Chaduvula K, Priya R. Alzheimer's disease classification using competitive swarm multi-verse optimizer-based deep neuro-fuzzy network. Concurr Comput Pract Exp. 2023;35(21):1–19.

3. Mohi ud din dar G, Bhagat A, Ansarullah SI, Othman MT Ben, Hamid Y, Alkahtani HK, et al. A Novel Framework for Classification of Different Alzheimer's Disease Stages Using CNN Model. Electron. 2023;12(2):1–14.

4. Tufail A Bin, Anwar N, Othman MT Ben, Ullah I, Khan RA, Ma Y-K, et al. Early-Stage Alzheimer's Disease Categorization Using PET Neuroimaging Modality and Convolutional Neural Networks in the 2D and 3D Domains. Sensors [Internet]. 2022 Jun 18;22(12):4609. Available from: https://www.mdpi.com/1424-8220/22/12/4609

Reviewer 4, Comment # 11: Authors have addressed the comments made by previous reviewer; however, I would like to suggest that authors should update the literature review with recent studies from 2023, for improved readability and relevancy.

Response: Thank you for your comment and suggestion. Latest models, such as Hridhee et al. (2023), Allada et al. (2023), Gowhar et al. (2023) and Tufail et al. (2022), have been added.

Thank you.

Yours sincerely,

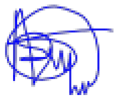

Mr. Nicodemus Songose Awarayi
Corresponding Author
nicodemus.awarayi@uenr.edu.gh
+233247242882
