# Supplementary material for: A bilateral filtering-based image enhancement for Alzheimer disease classification using CNN
Source: PLoS One. 2024 Apr 19;19(4):e0302358. doi: 10.1371/journal.pone.0302358 (PMC11029622; doi:10.1371/journal.pone.0302358)
Supplement: Attachment — Submitted filename: Response to reviewers.pdf [file pone.0302358.s004.pdf]

8th February, 2024.

The Editor

PLOS ONE Journal

Dear Sir/Madam,

Response to Review Comments

I wish to extend my gratitude to the editors and reviewers for their insightful feedback on our manuscript. We have diligently addressed all the comments and made the necessary changes to the manuscript. For your convenience, major revisions have been highlighted in yellow. Detailed responses to the reviewers' comments are provided below:

Manuscript Number: PONE-D-23-12558

Title: A Bilateral Filtering-based Image Enhancement for Alzheimer's Disease Classification Using CNN

Reviewer 4, Comment #1: The authors have addressed all the comments in an adequate manner. Therefore, I would like to recommend the acceptance of this article.

Response: Thank you for reviewing and accepting our manuscript.

Reviewer 5, Comment #1: The author has basically solved my problem, and I have no other problems. Now I recommend receiving the revised manuscript.

Response: Thank you for reviewing and accepting our manuscript.

Reviewer 6, Comment #1: The article entitled " A Bilateral Filtering-based Image Enhancement for Alzheimer Disease Classification using CNN " can be Accepted in its current form

Response: Thank you for reviewing and accepting our manuscript.

Reviewer 7, Comment #1: The overall representation/structure of paper is not appropriate. It needs figurative approach that should be representation of the overall proposed work.

Response: Thank you for the comment. We have reviewed the manuscript to ensure it follows the general guidelines of the PLOS ONE journal. We have also introduced more figures to improve the visual elements in the work for better clarity.

Reviewer 7, Comment #2: With necessary images show the impact of proposed enhancement techniques (histogram equalization, bilateral filtering).

Response: Thank you for the suggestion. Images of the proposed enhancement techniques are presented in the Fig 3 of the manuscript. A further comparative assessment of the image quality was performed based on mean pixel intensity, contrast and entropy metrics and presented in Fig 4 of the manuscript.

Reviewer 7, Comment #3: Describe with necessary data how the preprocessing techniques improves the overall accuracy of the proposed work.

Response: Table 2 of the manuscript has been modified to include the test results of the model before the image enhancement.

Reviewer 7, Comment #4: Compare the result with the standard CNN models like VGG19, ResNet110, Dense net also compare the number of parameters.

Response: Thank you for your recommendation. This comparison has already been done in Table 4 of the manuscript. The study by Hridhee et al., (2023) used VGG16 model while Zhang et al., (2022) used the ResNet110 model.

Reviewer 7, Comment #5: Data samples are imbalanced how this is handled please describe.

Response: Your comments are duly appreciated. Data augmentation was used to increase the data volume; however, in the case of data imbalance from the MCI class, it was observed not to be significant since it did not affect the training process. This can be seen in the confusion matrices in Figs 5 and 6 of the manuscript.

Reviewer 7, Comment #6: Show the confusion matrix for each class and also describe the ROC curves..

Response: Thank for the comment. The confusion matrix and the ROC curves have been included. Refer to the manuscript Figs 5, 6 and 7.

Thank you.

Yours sincerely,

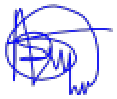

Mr. Nicodemus Songose Awarayi
Corresponding Author
nicodemus.awarayi@uenr.edu.gh
+233247242882
